# Supplementary material for: Large-scale genome-wide study reveals climate adaptive variability in a cosmopolitan pest
Source: Nat Commun. 2021 Dec 10;12:7206. doi: 10.1038/s41467-021-27510-2 (PMC8664911; doi:10.1038/s41467-021-27510-2)
Supplement: Supplementary file 1 — Supplementary information [file 41467_2021_27510_MOESM1_ESM.pdf]

Supplementary information for

Large-scale genome-wide study reveals climate adaptive variability in a cosmopolitan pest

Yanting Chen[1,2,3,4], Zhaoxia Liu[1,2,3,5], Jacques Régnière[6], Liette Vasseur[1,2,7], Jian Lin[8], Shiguo Huang[8], Fushi Ke[1,2,3,9], Shaoping Chen[1,2,3,4], Jianyu Li[1,2,3,4], Jieling Huang[1,2,3], Geoff M. Gurr[1,2,10*], Minsheng You[1,2,3*], Shijun You[1,2,3*]

Supplementary Figures and Tables

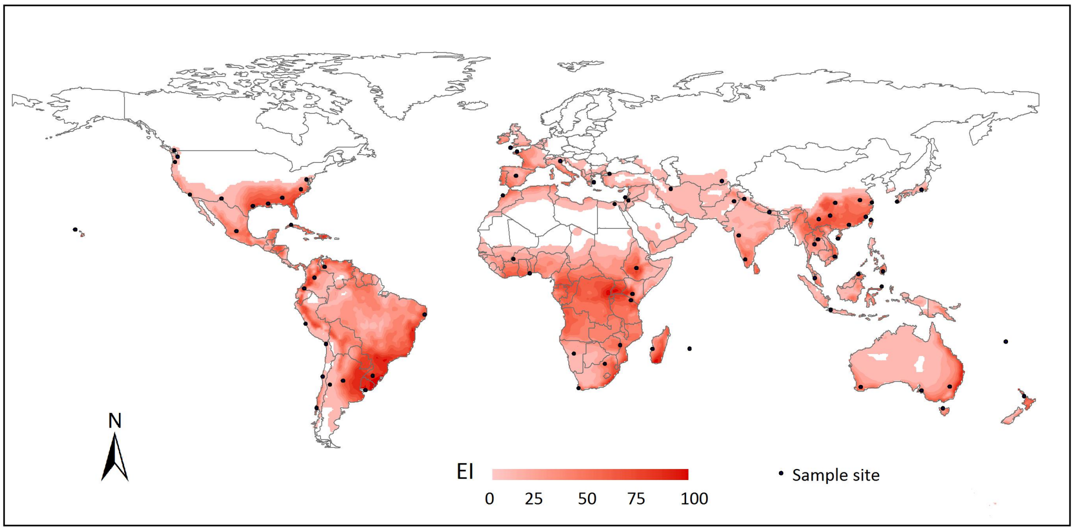

Supplementary Figure 1. Map of sampling sites showing the locations where specimens were collected (see Supplementary Data 1 for details) across year-round persistence regions of diamondback moth in the world. A total of 372 specimens were collected from 78 sampling sites in the year-round persistence regions of diamondback moth across the world, with 13 samples from Africa, 29 from Asia, 5 from Europe, 13 from North America including Hawaii, 12 from South America, and 6 from Oceania. The red-shaded range represents the year-round persistence regions of diamondback moth (EI > 0).

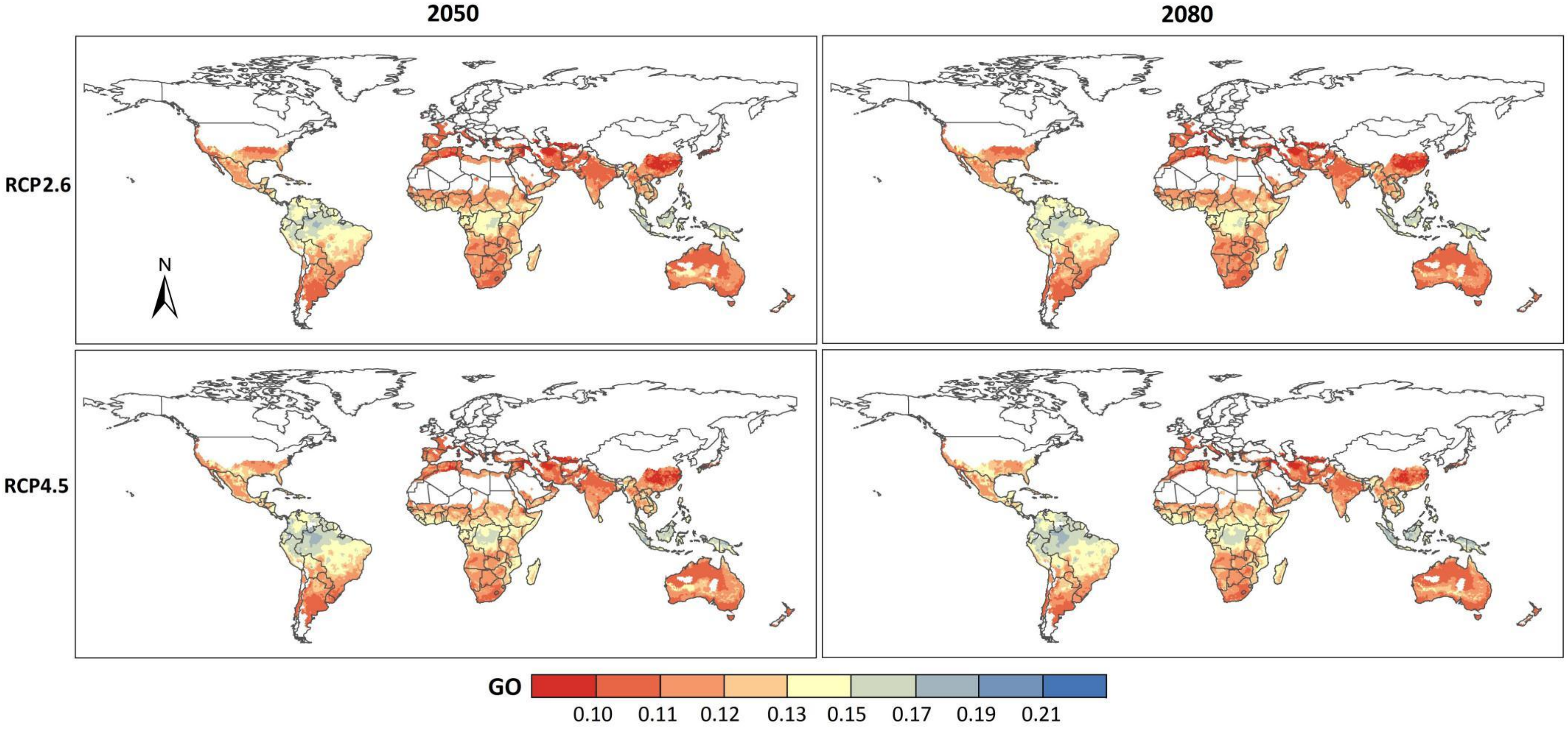
2050
2080
RCP2.6
RCP4.5
N
GO
0.10
0.11
0.12
0.13
0.15
0.17
0.19
0.21

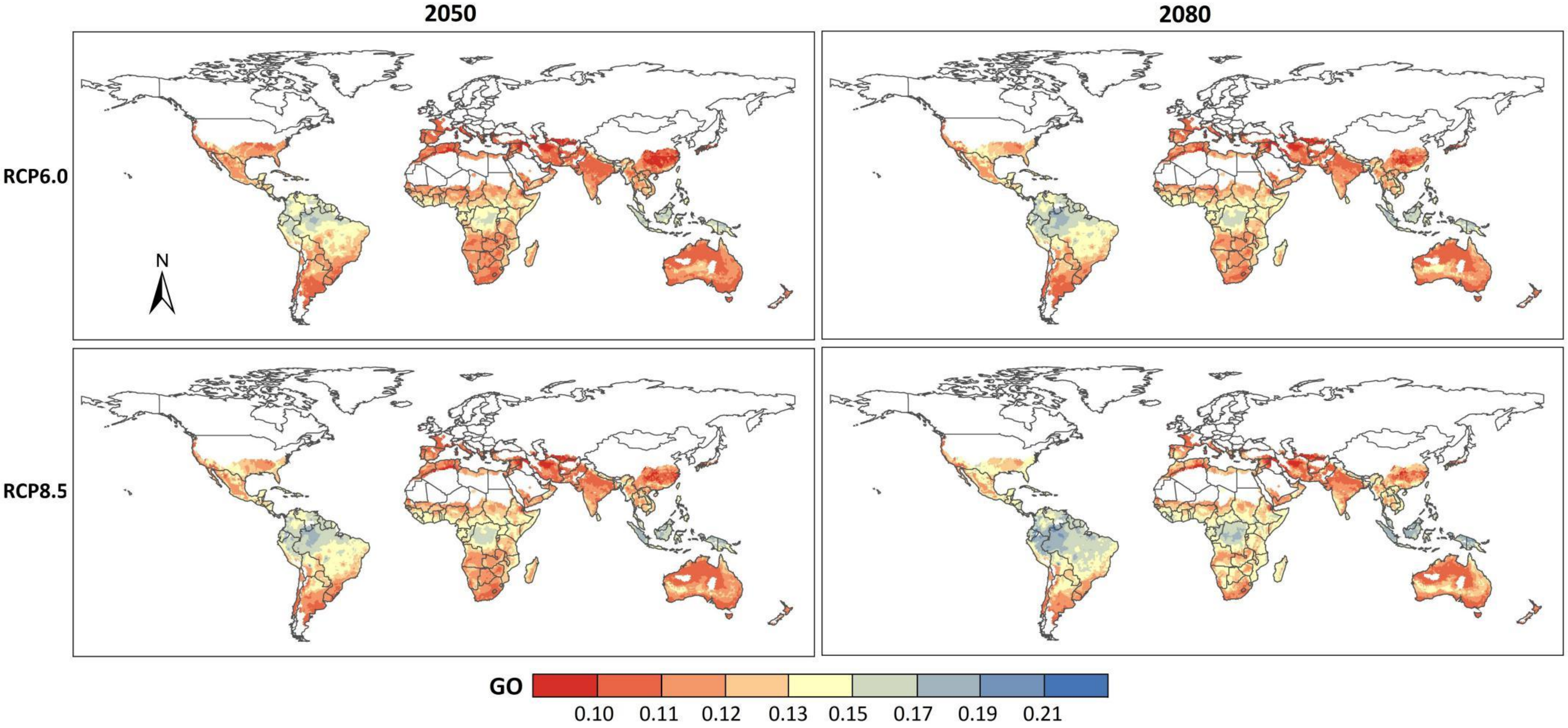

Supplementary Figure 2. Predicted distribution of genetic offset (GO) under four projected greenhouse gas emission scenarios RCP2.6, RCP4.5, RCP6.0 and RCP8.5 in 2050 and 2080 based on generalized dissimilarity modelling (GDM).

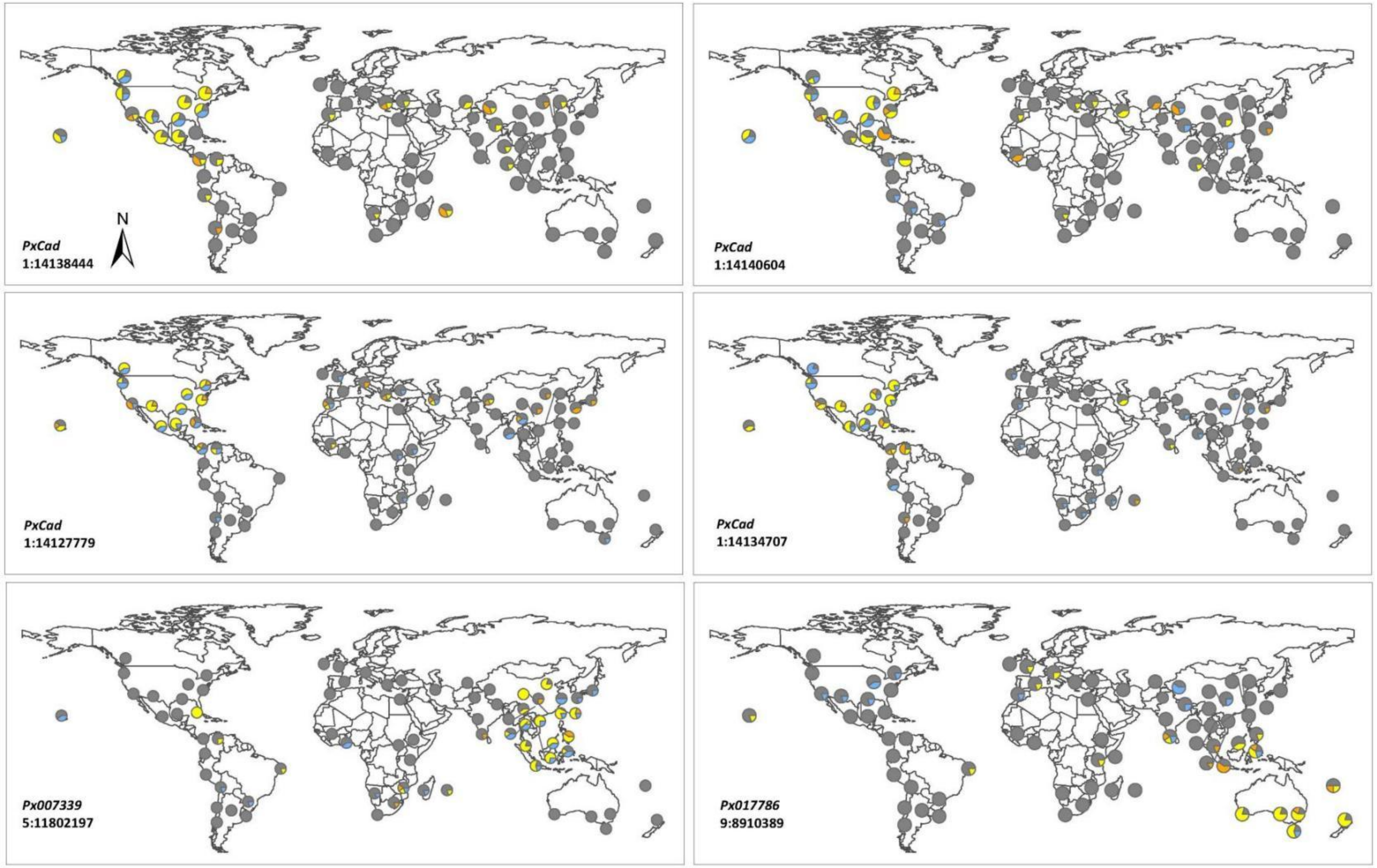

Supplementary Figure 3. Genomic signals of adaptive selection shown by the frequency distribution of four SNPs identified from the coding region of *PxCad* and two SNPs associated with the two previously reported temperature-related genes (Px007339 and Px017786). The colors represent: grey: original genotypes; yellow: homozygous mutants; orange: heterozygous mutants, and blue: missing sites, respectively.

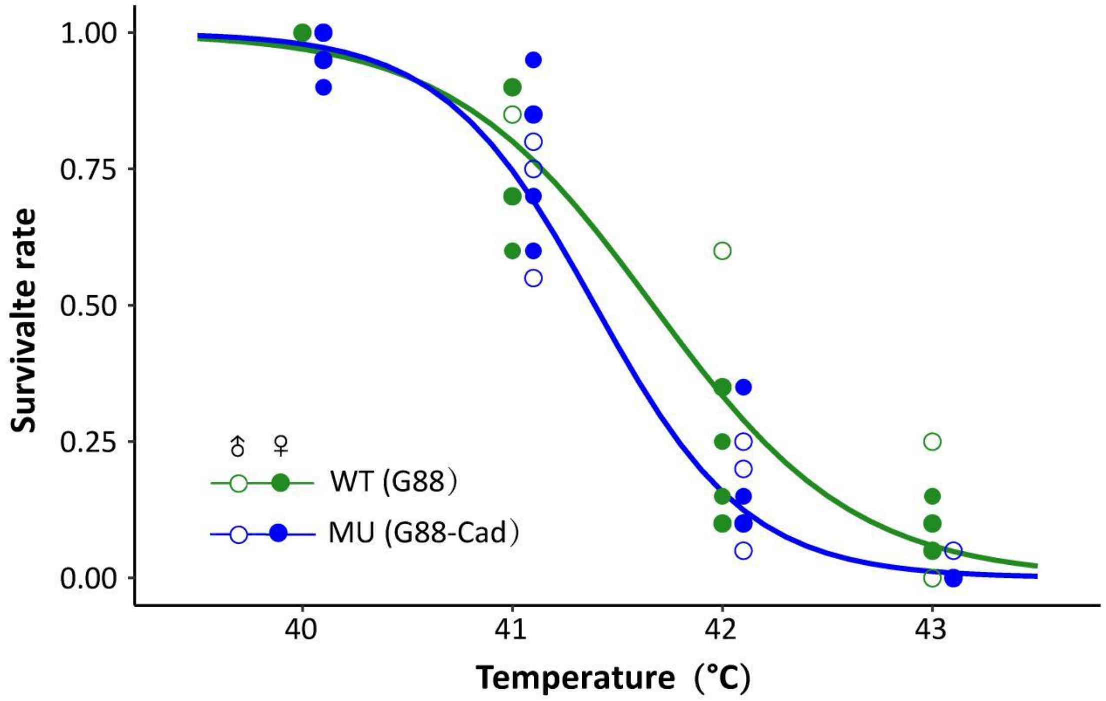

Supplementary Figure 4. Relationship between survival rate and high temperatures using males and females of wild-type (WT, green) and *PxCad*-deficient (MU, blue) diamondback moth strains. Dots represent observed survival of 20 individuals with four replicates for each of the treatments, and solid lines represent the fitted survival rates from the equations in Supplementary Table 4.

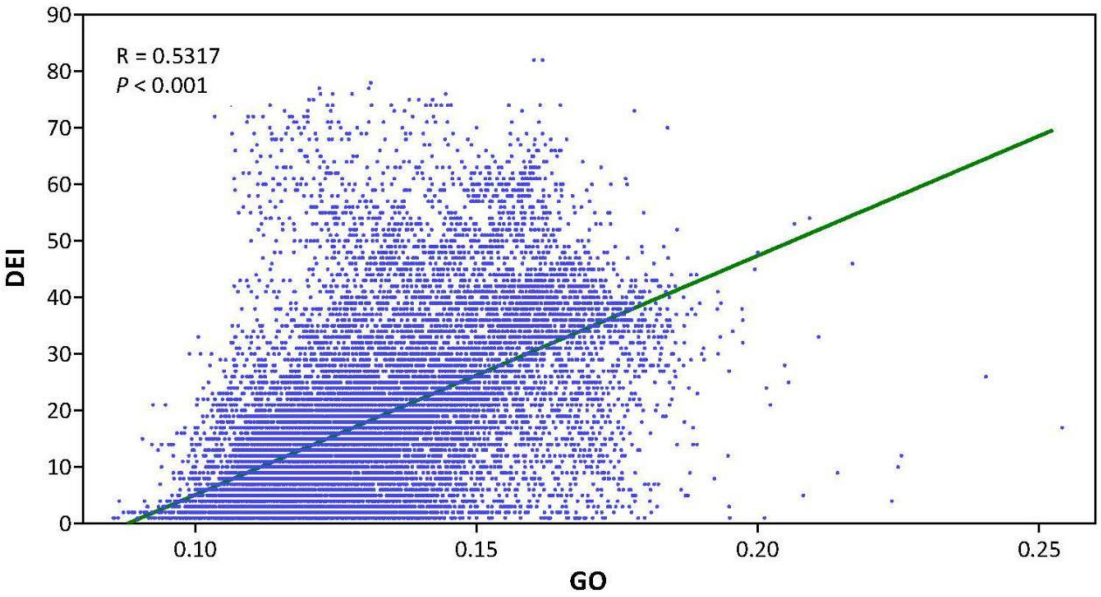

Supplementary Figure 5. Correlation between GO and DEI based on the Pearson Correlation Coefficient.

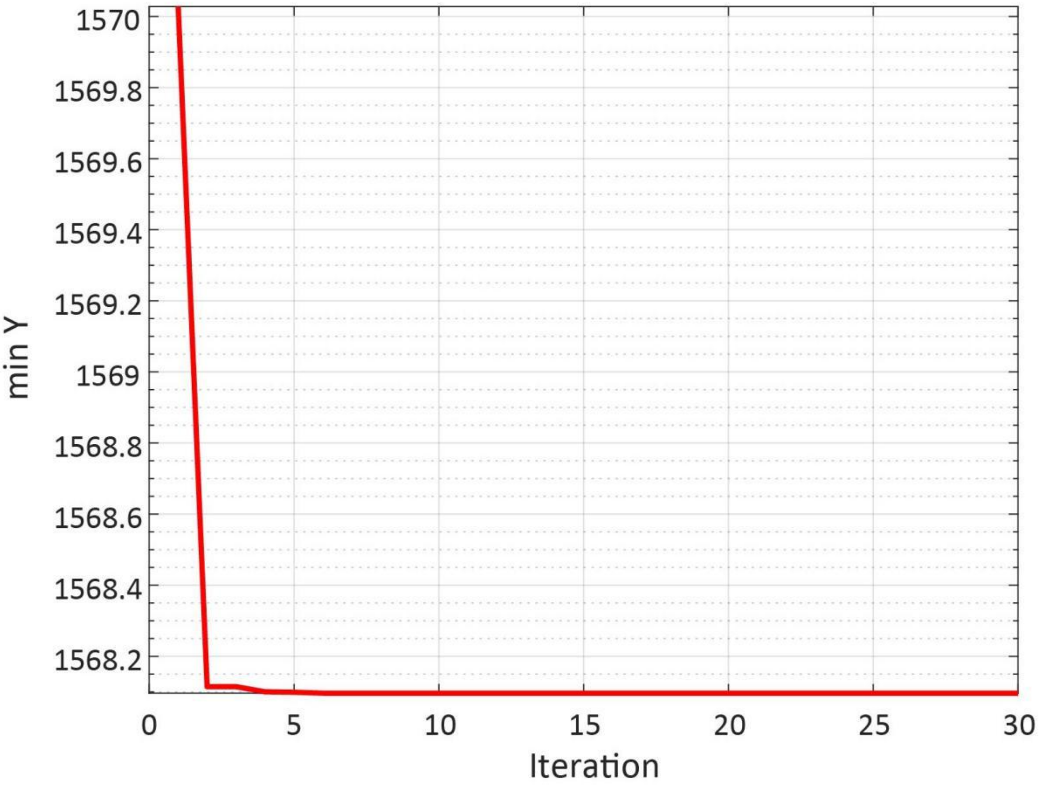

Supplementary Figure 6. The convergence curve of the ABC algorithm.

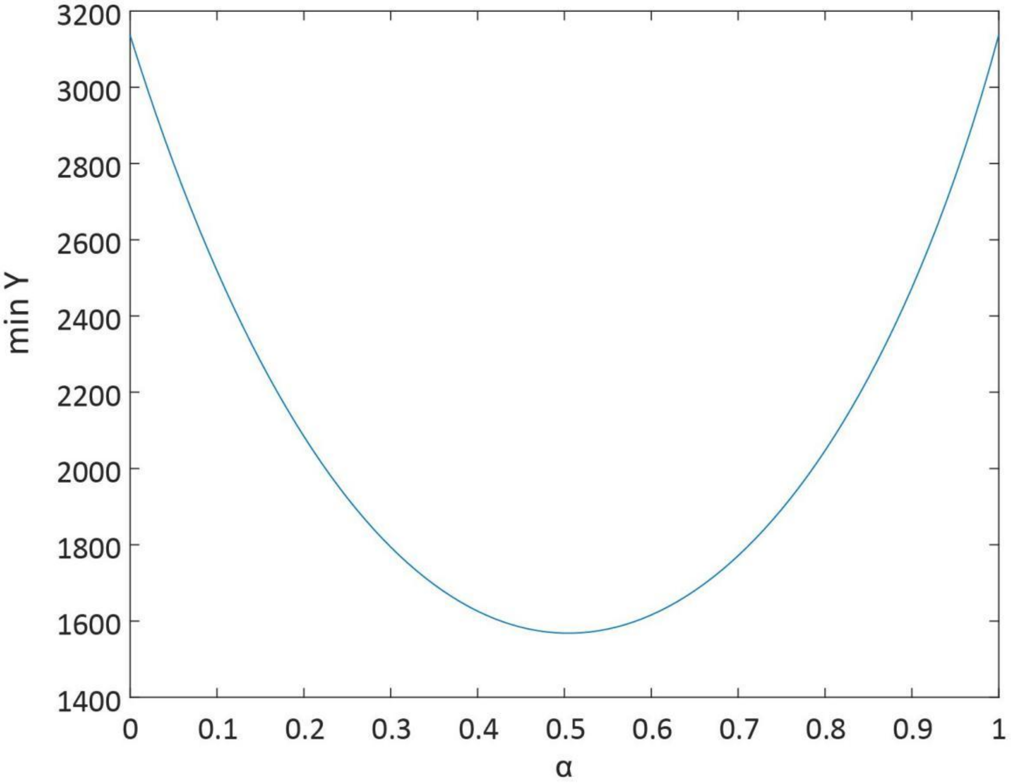

Supplementary Figure 7. The function curve of α and Y.

Supplementary Table 1. Summary of climate variables used for testing the correlations between genomic variation and climate conditions.

Variables	Description
Bio1	Annual Mean Temperature
Bio2	Mean Diurnal Range (Mean of monthly (max temp - min temp))
Bio3	Isothermality (Bio2/Bio7) (* 100)
Bio4	Temperature Seasonality (standard deviation *100)
Bio5	Max Temperature of Warmest Month
Bio6	Min Temperature of Coldest Month
Bio7	Temperature Annual Range (Bio5-Bio6)
Bio8	Mean Temperature of Wettest Quarter
Bio9	Mean Temperature of Driest Quarter
Bio10	Mean Temperature of Warmest Quarter
Bio11	Mean Temperature of Coldest Quarter
Bio12	Annual Precipitation
Bio13	Precipitation of Wettest Month
Bio14	Precipitation of Driest Month
Bio15	Precipitation Seasonality (Coefficient of Variation)
Bio16	Precipitation of Wettest Quarter
Bio17	Precipitation of Driest Quarter
Bio18	Precipitation of Warmest Quarter
Bio19	Precipitation of Coldest Quarter

Supplementary Table 2. Summary of the 94 putatively adaptive SNPs collectively detected by at least two of the three models (Samβada (S), LFMM (L), and Bayenv 2 (B)).

SNP	Gene ID (gene name)	Genomic region	Model	bio1	bio2	bio3	bio4	bio5	bio6	bio7	bio8	bio9	bio 10	bio 11	bio 12	bio 13	bio 14	bio 15	bio 16	bio 17	bio 18	bio 19	reference
1:14140604	Px012847 (*PxCad*)	Exon	S, L, B*		△○□ **					□													
1:14138444			S, L, B		△○□				□														
8:17959406	-	Intergenic	S, L, B		△□				□	□	○		△										
17:4166692	-	Intergenic	S, L, B		△○□					△													
1:19367073	-	Intergenic	S, L, B		○			△	□	□				□									
1:14127779	Px012847	Exon	S, B		□					△													
1:14134707	Px012847	Exon	S, B		△□																		
13:6927806	Px006020	Exon	S, B			△□			□		□			□	□	□			□				
5:11815334	Px007340	Exon	S, B	□					△□	□				□									
5:6973906	Px005423	Exon	S, B								□					□			□		□		
6:13269140	Px014654	Exon	S, B	□										□		□			□				
9:5341286	Px005767	Exon	S, B					△					△ □										
1:1357238	Px003712	Exon	S, B					△					△									□	
1:18271204	Px005886	Exon	S, B					△□					□										
17:15419750	Px012020	Exon	S, B								□										□		
5:11802197	Px007339	Exon	S, B	□					△□		□			□									1, 2
5:6965337	Px005422	Exon	S, B								□					□			□		□		
1:1357580	Px003712	Exon	S,B					△					△ □										

SNP	Gene ID (gene name)	Genomic region	Model	bio1	bio2	bio3	bio4	bio5	bio6	bio7	bio8	bio9	bio 10	bio 11	bio 12	bio 13	bio 14	bio 15	bio 16	bio 17	bio 18	bio 19	reference
1:19322492	Px005926	Exon	S,B		△				□	□													
13:1607687	Px005956	Exon	S,B	□				△					△	□									
1:19166864	Px005917	Exon	L, B	○	□				○	○	○			○									
1:19199374	Px005920	Exon	L,B	○	○		○		○	○□	○			○		○					○		
1:19321262	Px005926	Exon	L,B		○□		○		○	○				○									
11:14729263	Px003623	Exon	L,B												○		○					□	
9:2482427	Px016903	Exon	L,B		○□																		
9:143305	Px016688	Intron	S, L		△						○		△										
11:17356902	Px004050	Intron	S, B					△			□		△□										
5:11842782	Px007342	Intron	S, B	□			□		□	□	□			□									
5:11845554	Px007342	Intron	S, B	□					△□	□	□			□									
5:11848381	Px007342	Intron	S, B	△□					□	□	□			△□									
1:273656	Px003665	Intron	S, B				□			△□													
1:7225560	Px003267	Intron	S,B		△□				□	□													
1:8077334	Px003207	Intron	S,B				△□		□	□													
9:8910389	Px017786	Intron	S,B					△□					△□										3, 4
12:19772797	Px005362	Intron	S,B		△□																		
12:19777368	Px005362	Intron	S,B		△□																		
13:6930159	Px006021	Intron	L, B	□		□	○		○					○□									

SNP	Gene ID (gene name)	Genomic region	Model	bio1	bio2	bio3	bio4	bio5	bio6	bio7	bio8	bio9	bio 10	bio 11	bio 12	bio 13	bio 14	bio 15	bio 16	bio 17	bio 18	bio 19	reference
3:2658494	Px007104	Intron	L, B														○□			○□			
1:14295167	Px012858	Intron	L,B	○	○□				○		○			○									
1:19193140	Px005920	Intron	L,B	○	○□		○		○□	○	○			○									
1:7176982	Px003270	Intron	L,B		○□					○													
6:5927131	Px003314	Intron	L,B	○		□			○		○			○									
13:14488412	-	Intergenic	S, L	○				△			○		○										
1:3541169	-	Intergenic	S, L		△												○			○			
12:11605107	-	Intergenic	S, L					△					△								○		
13:11552881	-	Intergenic	S, L					△					△			○					○		
13:11552906	-	Intergenic	S, L					△								○					○		
13:7756289	-	Intergenic	S, L					△	○				△	○									
8:10036046	-	Intergenic	S, L					△								○							
12:1147665	-	Intergenic	S, B	□					△□			□	□	△□									
13:10703881	-	Intergenic	S, B		△□				□	△													
13:5359926	-	Intergenic	S, B					△□			□		△□										
16:19439907	-	Intergenic	S, B					△□					□										
5:11820699	-	Intergenic	S, B	□					△□	□				□									
5:11862353	-	Intergenic	S, B	□					□					□									
5:4410220	-	Intergenic	S, B					△							□	□			□				
5:6971473	-	Intergenic	S, B													△□			□		□		

SNP	Gene ID (gene name)	Genomic region	Model	bio1	bio2	bio3	bio4	bio5	bio6	bio7	bio8	bio9	bio 10	bio 11	bio 12	bio 13	bio 14	bio 15	bio 16	bio 17	bio 18	bio 19	reference
5:6971499	-	Intergenic	S, B													□			□		□		
5:6976799	-	Intergenic	S, B	△												□			□		□		
9:3425415	-	Intergenic	S, B	△	□																		
10:9197738	-	Intergenic	S,B		△□																		
13:7758227	-	Intergenic	S,B					△□					△□										
13:14452088	-	Intergenic	S,B	□				△					△										
13:6935702	-	Intergenic	L,B	○					○□					○	○	○			○		○		
16:6153153	-	Intergenic	L,B		○□					○													
16:6153291	-	Intergenic	L,B		○										□	□			□		○□		
2:10846394	-	Intergenic	L,B						○					○							□		
6:5771117	-	Intergenic	L,B	○					○		○□			○					○				
8:17771220	-	Intergenic	L,B		○□																		
8:17959823	-	Intergenic	L,B	○	○□		○		○	○	○			○							○		
9:2414701	-	Intergenic	L,B		○□				○	○													
9:2611378	-	Intergenic	L,B		○				□														
9:2628608	-	Intergenic	L,B		○				○□	○													
8:5615011	-	Intergenic	L,B					○								□			□			□	
1:11336364	-	Intergenic	L, B		○									□									
1:19419094	-	Intergenic	L, B	○	□																		
1:15128513	-	Intergenic	L,B									○□		○									
1:19319356	-	Intergenic	L,B		○				○□	○				○□		○					○		

SNP	Gene ID (gene name)	Genomic region	Model	bio1	bio2	bio3	bio4	bio5	bio6	bio7	bio8	bio9	bio 10	bio 11	bio 12	bio 13	bio 14	bio 15	bio 16	bio 17	bio 18	bio 19	reference
1:5621916	-	Intergenic	L,B				□								○	○			○			○	
1:7202452	-	Intergenic	L,B		○□					○													
10:9198357	-	Intergenic	L,B		□				○														
11:3706230	-	Intergenic	L,B	○					○					○<br>□									
11:7905763	-	Intergenic	L,B			□					○												
13:19137327	-	Intergenic	L,B								□										○		
13:6935544	-	Intergenic	L,B	○		○□	○□		○	○	○			○	○								
1:19518653	-	Intergenic	L, B	○	□						○		○										
1:19544609	-	Intergenic	L, B	□	○				○	○													
10:9197915	-	Intergenic	L, B	○					○□	□	○			○									
13:6938168	-	Intergenic	L, B	□		□	□		□	□	○□			○<br>□									
13:6939736	-	Intergenic	L, B			○	○			○	□			○									
14:17278647	-	Intergenic	L, B	□	○				□	□				□									
14:17288941	-	Intergenic	L, B		○□					○					○	○					○		
5:15404119	-	Intergenic	L, B		○□				○	○													
6:6056198	-	Intergenic	L, B			□	□			○													
Total loci				31	40	8	13	20	40	35	27	2	19	33	8	18	3	0	13	2	17	4	

*S, L, and B indicates that the SNP was identified by Samβada (S), LFMM (L), and Bayenv 2 (B) to be correlated with climate variables. The top five SNPs were identified by all three models (S, L, and B).

**△, ○, or/and □ indicates a given climate variable was detected by Samβada, LFMM, or/and Bayenv 2 to be associated with the corresponding SNP on the left column. Gene with reference number indicate those genes being previously documented as having temperature-related functions.

Supplementary Table 3. Logistic regression analysis of survival responses of both sexes of wild-type (WT, G88) and *PxCad*-deficient mutant (MU, G88-Cad) strains to cold-shock treatments. Equation: log[*p*/(1-*p*)] = *a* + *b t* where *t* is duration (min). Wald χ^2 statistic.

		-14°C		-17°C		-20°C	
**Source**	**DF**	**χ^2**	**P**	**χ^2**	**P**	**χ^2**	**P**
Regression	7	194.9	< 0.001	322.06	< 0.001	290.75	< 0.001
Duration	1	60.51	< 0.001	82.58	< 0.001	78.57	< 0.001
Strain	1	5.67	0.017	15.81	< 0.001	37.04	< 0.001
Sex	1	3.90	0.048	0.03	0.856	0.74	0.391
Duration×Strain	1	4.00	0.045	0.15	0.695	31.29	< 0.001
Duration×Sex	1	19.36	0.002	0.25	0.619	0.79	0.374
Strain×Sex	1	4.96	0.026	0.55	0.458	2.42	0.120
Duration×Strain*Sex	1	9.74	0.002	0.06	0.810	3.37	0.066
Wild-type (WT)	Males	4.181 – 0.01628 *t*		7.506 – 0.08923 *t*		16.07 – 0.5178 *t*	
	Females	2.730 – 0.007566 *t*		7.729 – 0.08209 *t*		14.04 – 0.4495 *t*	
Mutant (MU)	Males	2.511 – 0.01070 *t*		3.444 – 0.08324 *t*		4.397 – 0.1654 *t*	
	Females	3.267 – 0.01425 *t*		4.773 – 0.08104 *t*		6.374 – 0.2541 *t*	

Supplementary Table 4. Logistic regression analysis of survival responses of both sexes of wild-type (WT, G88) and *PxCad*-deficient mutant (MU, G88-Cad) strains to heat-shock treatments. Equation: log[*p*/(1-*p*)] = *a* + *b T* where *T* is temperature (°C). Wald χ^2 statistic. Parameter values estimates for both sexes pooled.

**Source**	**DF**	**χ^2**	**P**
Regression	7	370.56	< 0.001
Temperature	1	94.37	< 0.001
Strain	1	4.79	0.029
Sex	1	0.21	0.647
Temperature×Strain	1	5.03	0.025
Temperature×Sex	1	0.23	0.629
Strain×Sex	1	0.32	0.571
Temperature×Strain×Sex	1	0.34	0.557
Wild-type (WT)	Males & females	86.71 - 2.081 *T*	
Mutant (MU)	Males & females	114.2 - 2.759 *T*	

Supplementary Table 5. Pairwise Pearson correlation coefficient between climate variables.

	Bio01	Bio02	Bio03	Bio04	Bio05	Bio06	Bio07	Bio08	Bio09	Bio10	Bio11	Bio12	Bio13	Bio14	Bio15	Bio16	Bio17	Bio18	Bio19
Bio01	-	-0.09	0.49	-0.48	0.67	0.86	-0.41	0.77	0.65	0.80	0.92	0.39	0.45	0.14	0.21	0.45	0.14	0.35	0.16
Bio02		-	-0.02	0.37	0.44	-0.44	0.67	0.09	-0.20	0.14	-0.24	-0.43	-0.29	-0.35	0.37	-0.32	-0.37	-0.21	-0.40
Bio03			-	-0.87	-0.09	0.69	-0.70	0.31	0.40	-0.05	0.73	0.44	0.32	0.33	0.03	0.36	0.34	0.24	0.42
Bio04				-	0.29	-0.82	0.93	-0.22	-0.47	0.14	-0.79	-0.44	-0.36	-0.27	-0.04	-0.40	-0.28	-0.24	-0.40
Bio05					-	0.20	0.40	0.67	0.28	0.94	0.33	-0.01	0.15	-0.13	0.29	0.11	-0.15	0.15	-0.21
Bio06						-	-0.82	0.57	0.65	0.41	0.97	0.53	0.50	0.30	0.05	0.52	0.30	0.36	0.37
Bio07							-	-0.14	-0.45	0.17	-0.71	0.50	-0.38	-0.36	0.12	-0.42	-0.37	-0.25	-0.47
Bio08								-	0.07	0.70	0.63	0.36	0.46	0.11	0.25	0.45	0.10	0.51	-0.04
Bio09									-	0.43	0.68	0.17	0.16	0.05	0.05	0.17	0.06	-0.06	0.29
Bio10										-	0.49	0.13	0.24	-0.02	0.18	0.22	-0.04	0.19	-0.08
Bio11											-	0.47	0.47	0.22	0.15	0.49	0.22	0.33	0.30
Bio12												-	0.84	0.79	-0.32	0.88	0.81	0.80	0.70
Bio13													-	0.38	0.13	0.99	0.41	0.86	0.38
Bio14														-	-0.69	0.44	1.00	0.48	0.80
Bio15															-	0.07	-0.70	0.01	-0.51
Bio16																-	0.47	0.87	0.43
Bio17																	-	0.50	0.80
Bio18																		-	0.28
Bio19																			-

Supplementary Table 6. Description of CLIMEX indices and parameters used for prediction of habitat suitability of diamondback moth.

Index	Parameters	Description	Value
Temperature	DV0	Limiting low temperature	7℃
	DV1	Lower optimal temperature	14℃
	DV2	Upper optimal temperature	28℃
	DV3	Limiting high temperature	38℃
	PDD	Degree-days per generation	268 Degree Days
Moisture	SM0	Limiting low soil moisture	0.05%
	SM1	Lower optimal soil moisture	0.5%
	SM2	Upper optimal soil moisture	1.25%
	SM3	Limiting high soil moisture	1.75%
Cold stress	DTCS	Threshold number of degree-days below DV0 at which cold stress begins to accumulate.	12 Degree Days
	DHCS	Rate at which cold stress accumulates when the threshold number of degree-days below DV0 (DTCS) is reached	-0.0005
	TTCS	Cold stress temperature threshold	4℃
	THCS	Cold stress temperature rate	-0.0005/℃/day
Heat stress	TTHS	Heat stress temperature threshold	38℃
	THHS	Heat stress temperature rate	0.001/℃/day
	DTHS	Threshold number of degree-days above DV3 at which heat stress begins to accumulate.	1 Degree Days
	DHHS	Rate at which heat stress accumulates when the threshold number of degree-days above DV0 (DTHS) is reached	0.005
Dry stress	SMDS	Dry stress threshold	0.05%
	HDS	Dry stress rate	-0.005/day
Wet stress	SMWS	Wet stress threshold	1.75%
	HWS	Wet stress rate	0.05/day
Hot-Wet stress	TTHW	Temperature at which hot-wet stress begins	32℃
	MTHW	Soil moisture at which hot-wet stress begins	1.25%
	PHW	Rate at which hot-wet stress accumulates once Tmax > TTHW & soil moisture > MTHW	0.05

Supplementary Table 7. Primers used for *PxCad* study.

Primer	Sequence (5′-3′)	Direction	Application
Cad-sg1_F	5′-TAATACGACTCACTATAGGAGGGAGATGTCATCATTGCAGTTTTAGAGCTAGAAATAGCAAGTTAA-3′[*]	Sense	PCR amplification of the template for *in vitro* transcription of the sgRNA of Cad-sg1.
Cad-sg2_F	5′-TAATACGACTCACTATAGGACTCGTCCATGTAGATGTTGGTTTTAGAGCTAGAAATAGCAAGTTAA-3′[*]	Sense	PCR amplification of the template for *in vitro* transcription of the sgRNA of Cad-sg2.
sgRNA_comR	5′-AAAAGCACCGACTCGGTGCCACTTTTTCAAGTTGATAACGGACTAGCCTTATTTTAACTTGCTATTTCTAGCTCTAAAA-3′	Antisense	PCR amplification of the template for *in vitro* transcription of the sgRNAs of Cad-sg1 and Cad-sg2.
8fPxCad	5′-CATCGGAYCCATGTCTCAG-3′	Sense	PCR amplification to detect the mutation generated by CRISPR/Cas9
7rPxCad	5′-TTCTGTGTAATGACGAGGTG-3′	Antisense	PCR amplification to detect the mutation generated by CRISPR/Cas9

[*]Forward primer containing the T7 polymerase binding site and the underlined sgRNA target sequence.

References

1. Huang, H. J. et al. Comparative analysis of the transcriptional responses to low and high temperatures in three rice planthopper species. *Mol. Ecol.* **26**, 2726-2737 (2017).
2. Liu, Y. C. et al. Comparative transcriptome analysis of *Glyphodes pyloalis* Walker (Lepidoptera: Pyralidae) reveals novel insights into heat stress tolerance in insects. *BMC Genomics* **18**, 974 (2017).
3. Lansdell, S. J., Schmitt, B., Betz, H., Sattelle, D. B., Millar, N. S. Temperature-sensitive expression of *Drosophila neuronal* nicotinic acetylcholine receptors. *J Neurochem* **68**, 1812-1819 (1997).
4. Nie, H. Y. et al. Transcriptome Analysis of neonatal larvae after hyperthermia-induced seizures in the contractile silkworm, *Bombyx mori*. *PloS One* **9**, e113214 (2014).
