# Supplementary material for: Large-scale genome-wide study reveals climate adaptive variability in a cosmopolitan pest
Source: Nat Commun. 2021 Dec 10;12:7206. doi: 10.1038/s41467-021-27510-2 (PMC8664911; doi:10.1038/s41467-021-27510-2)
Supplement: Supplementary file 3 — Description of Additional Supplementary Files [file 41467_2021_27510_MOESM3_ESM.pdf]

Supplementary Data legends

File Name: Supplementary Data 1

Description:Summary of the diamondback moth samples used for this study.

File Name: Supplementary Data 2

Description: Sequencing Statistics of 372 diamondback moth individuals.

File Name: Supplementary Data 3

Description: Identification of the SNPs associated with climate variables using Samβada.

File Name: Supplementary Data 4

Description: Identification of the SNPs associated with climate variables using LFMM.

File Name: Supplementary Data 5

Description: Identification of the SNPs associated with climate variables using Bayenv 2.
