# Supplementary material for: Large-scale genome-wide study reveals climate adaptive variability in a cosmopolitan pest
Source: Nat Commun. 2021 Dec 10;12:7206. doi: 10.1038/s41467-021-27510-2 (PMC8664911; doi:10.1038/s41467-021-27510-2)
Supplement: Supplementary file 5 — Reporting summary [file 41467_2021_27510_MOESM5_ESM.pdf]

nature research

Corresponding author(s): Shijun You

Last updated by author(s): Nov 10, 2021

Reporting Summary

Nature Research wishes to improve the reproducibility of the work that we publish. This form provides structure for consistency and transparency in reporting. For further information on Nature Research policies, see our Editorial Policies and the Editorial Policy Checklist.

Statistics

For all statistical analyses, confirm that the following items are present in the figure legend, table legend, main text, or Methods section.

n/a	Confirmed	
☐	☒	The exact sample size (n) for each experimental group/condition, given as a discrete number and unit of measurement
☐	☒	A statement on whether measurements were taken from distinct samples or whether the same sample was measured repeatedly
☐	☒	The statistical test(s) used AND whether they are one- or two-sided<br>*Only common tests should be described solely by name; describe more complex techniques in the Methods section.*
☐	☒	A description of all covariates tested
☐	☒	A description of any assumptions or corrections, such as tests of normality and adjustment for multiple comparisons
☐	☒	A full description of the statistical parameters including central tendency (e.g. means) or other basic estimates (e.g. regression coefficient) AND variation (e.g. standard deviation) or associated estimates of uncertainty (e.g. confidence intervals)
☐	☒	For null hypothesis testing, the test statistic (e.g. F, t, r) with confidence intervals, effect sizes, degrees of freedom and P value noted<br>*Give P values as exact values whenever suitable.*
☐	☒	For Bayesian analysis, information on the choice of priors and Markov chain Monte Carlo settings
☒	☐	For hierarchical and complex designs, identification of the appropriate level for tests and full reporting of outcomes
☐	☒	Estimates of effect sizes (e.g. Cohen's d, Pearson's r), indicating how they were calculated

Our web collection on statistics for biologists contains articles on many of the points above.

Software and code

Policy information about availability of computer code

Data collection	No softwares were used for data collection.
Data analysis	Publicly available softwares, including Stampy (v1.0.27), VCFtools (v.0.1.6), Sam.ada (v0.5.3), latent factor mixed model (LFMM) (v1.4), Bayenv 2, ArcGIS 10.2, and CLIMEX 2.0 are detailed in the section of Methods. The code of R package LEA was retrieved from http://membres-timc.imag.fr/Olivier.Francois/lfmm/files/note.pdf. The code of R package gdm was retrieved from https://cran.r-project.org/web/packages/gdm/gdm.pdf. The code of R package hierfstat was retrieved from https://mran.microsoft.com/web/packages/hierfstat/hierfstat.pdf.

For manuscripts utilizing custom algorithms or software that are central to the research but not yet described in published literature, software must be made available to editors and reviewers. We strongly encourage code deposition in a community repository (e.g. GitHub). See the Nature Research guidelines for submitting code & software for further information.

Data

Policy information about availability of data

All manuscripts must include a data availability statement. This statement should provide the following information, where applicable:

- Accession codes, unique identifiers, or web links for publicly available datasets
- A list of figures that have associated raw data
- A description of any restrictions on data availability

The genomic data that support the findings of this study have been deposited in the CNSA (https://db.cngb.org/cnsa/) of CNGBdb with accession code CNP0000018, and is synchronously accessible in the EBI-ENA (https://www.ebi.ac.uk/ena) with the accession code PRJEB24034. The climatic data were retrieved from WorldClim (https://www.worldclim.org).

Field-specific reporting

Please select the one below that is the best fit for your research. If you are not sure, read the appropriate sections before making your selection.

- [ ] Life sciences
- [ ] Behavioural & social sciences
- [x] Ecological, evolutionary & environmental sciences

For a reference copy of the document with all sections, see nature.com/documents/nr-reporting-summary-flat.pdf

Ecological, evolutionary & environmental sciences study design

All studies must disclose on these points even when the disclosure is negative.

Study description

Based on the globally-distributed nature of diamondback moth, we performed a landscape genomics study on our global samples.
For the gene expression analysis of PxCad, the treatment factor is temperature. Each treatment was repeated three times. We compared the gene expression between each of the temperature treatment and the control.
For the bioassay of behavioral responses to different temperatures, the treatment factor is the DBM strains. The observation of behavioral responses (i.e. survival rate) to different temperature setting was repeated four times for each DBM strains (i.e. wildtype strains vs. PxCad-deficient strain).

Research sample

For this study, without aiming to identify the differences between gender and age (developmental stages) in responding to climate change, Diamondback moths, Plutella xylostella (Lepidoptera: Plutellidae), regardless of age and sex, were collected from cruciferous vegetable fields in each sampling locations without any manipulations. Field-collected samples were morphologically inspected and genetically checked with COI sequences to confirm their identity. The samples were preserved in 95% alcohol at -80℃ prior to DNA extraction.
Samples used in this study represent local populations of the 75 collection sites.

Sampling strategy

Based on whole genome resequencing studies, especially on insect species, published previously, we followed their rules for sample size determination, i.e. five (fully sequenced) individuals per site should be good enough to give a robust 'picture' of the intra-population genomic variability as well as to compare and contrast with individual from other sites. The number of sites was set to give comprehensive coverage of all geographical regions in which this species is present, i.e. we tried our best to collect specimens as many as possible, given the availability of manpower and financial support.

Data collection

Within each sampling location, larvae, pupae, and adults were collected from cruciferous vegetable fields by our team members and local entomologists, inlcuding Hugo Cerda, Mark S. Goettel, Liette Vasseur, Geoff M. Gurr, Simon W. Baxter, Qisheng Song, Qinghai Fan, Gefu Wang-Pruski, David C. Lees, Jianlin Bai, Tiansheng Liu, Lu Peng, Miao Xie, Lijun Cai, Yunkai Zheng, Zhaohua Zeng, Sheng Lin, Yue Wang, Qian Zhao, Xiaofeng Xia, Wenbin Chen, Lilin Chen, Mingmin Zou, Jinying Liao, Liwei Han, Lianyun Lin, Yanping Lu, and Mousheng Zhuang. Record of sampling information was done by pen.

Timing and spatial scale

The sample collection of DBM started on November 5, 2012, and ended on August 8, 2014. Samples were collected from 114 locations that cover broad regions throughout the world, with 13 samples from Africa and Madagascar, 43 samples from Asia, 13 samples from Europe, 26 samples from North America including Hawaii, 12 samples from South America, and 7 samples from Oceania. For such a random sampling scheme, we only sampled once from each site. Our collection covered an extensive scope of the eco-climatic index and areas that support differing numbers of annual generations, including those regions with year-round persistence of DBM to others that are only seasonably suitable for growth and development of the species. In the present study, however, to investigate the adaptive genetic variation associated with contemporary climates, we used a subset of samples from regions in which DBM is able to persist year-round with a positive ecoclimatic index (EI > 0, where populations are subject to seasonally uninterrupted local selection by climatic factors.

Data exclusions

Before data analysis, we decided to remove the SNPs with a minor allele frequency (MAF) < 5% to generate common SNPs across the genome, based on the criteria applied in comparable studies. In addition, missing rate was high in sequencing data. We thus excluded the SNPs with a missing rate > 10% to retain majority of individuals and SNPs for further analysis, with the aim of reflecting a relatively complete picture of genomic variation.

Reproducibility

An average of approximately five individuals from each of the sampling locations were used for DNA extraction and sequencing, making a total of 372 individuals (with adequate quality data) in 78 locations worldwide. All attempts at replication in sampling, sequencing, gene expression analysis of PxCad, CRISPR/Cas9-based genome editing, bioassay (behavioral responses to different temperatures) were successful. We believe that our findings are convincing and reproducible.

Randomization

Our sampling locations were randomly selected in different regions according to the geographical and climatic conditions that are suitable for growth and development of DBM. Within each location, larvae, pupae and adults were randomly collected from cruciferous vegetable fields. In gene expression analysis of PxCad, a group of thirty vials (15 vials with DBM females and 15 with males) was frozen in liquid nitrogen and used as control, and other groups of vials were exposed to each of the nine distinct temperature treatments. After each of the treatments, moths were immediately frozen in liquid nitrogen. Each group of vials was divided into three replicates: both 15 vials with females and 15 with males were evenly put into three 1.5 ml tubes, respectively. In each replicates, the allocation was random. In CRISPR/Cas9-based genome editing experiment, the fresh eggs from the DBM strain G88, laid within 15 - 20 min, were injected. The DBM G88 individuals for laying eggs were chosen randomly. In the Bioassay: behavioral responses to different temperatures, twenty vials with females and twenty with males were then put into a plastic container. Four plastic containers with DBM adults were placed into a climate incubator where DBM adults were exposed to heat shock treatments. The allocation of female and male individuals to plastic containers was random, so was the allocation of plastic containers with DBM individuals to climate incubator.

Blinding	To avoid unintentional biases, the samples were each allocated a code number that was cryptic in not allowing anyone involved in handling or analysis to identify the origin of the insect, DNA or associated genomic data.

Did the study involve field work? ☐ Yes ☒ No

Reporting for specific materials, systems and methods

We require information from authors about some types of materials, experimental systems and methods used in many studies. Here, indicate whether each material, system or method listed is relevant to your study. If you are not sure if a list item applies to your research, read the appropriate section before selecting a response.

Materials & experimental systems

n/a	Involved in the study
☒	☐ Antibodies
☒	☐ Eukaryotic cell lines
☒	☐ Palaeontology and archaeology
☐	☒ Animals and other organisms
☒	☐ Human research participants
☒	☐ Clinical data
☒	☐ Dual use research of concern

Methods

n/a	Involved in the study
☒	☐ ChIP-seq
☒	☐ Flow cytometry
☒	☐ MRI-based neuroimaging

Animals and other organisms

Policy information about studies involving animals; ARRIVE guidelines recommended for reporting animal research

Laboratory animals	The study did not involve laboratory animals
Wild animals	The study did not involve wild animals.
Field-collected samples	Our field-collected samples were preserved in 95% alcohol and then stored at -80℃ freezer at the nearest entomological lab, prior to DNA extraction.
Ethics oversight	No ethical approval was required since our samples are insect pests.

Note that full information on the approval of the study protocol must also be provided in the manuscript.
